# Diagnostic Performance of Magnifying Endoscopy With Crystal Violet Staining for Superficial Non‐ampullary Duodenal Epithelial Tumors: A Single‐center Prospective Study

**DOI:** 10.1002/deo2.70223

**Published:** 2025-10-12

**Authors:** Tomo Kumei, Yosuke Toya, Shun Yamada, Makoto Eizuka, Shunichi Yanai, Masaki Endo, Ryo Sugimoto, Noriyuki Uesugi, Tamotsu Sugai, Naoki Yanagawa, Fumiaki Takahashi, Takayuki Matsumoto

**Affiliations:** ^1^ Department of Internal Medicine, Division of Gastroenterology and Hepatology, School of Medicine, Iwate Medical University Iwate Japan; ^2^ Kaiunbashi Endoscopy Clinic Iwate Japan; ^3^ Department of Molecular Diagnostic Pathology, School of Medicine Iwate Medical University Iwate Japan; ^4^ Diagnostic Pathology Center Southern Tohoku General Hospital Fukushima Japan; ^5^ Department of Information Science Division of Medical Engineering School of Medicine, Iwate Medical University Iwate Japan

**Keywords:** diagnosis, duodenal cancer, duodenal neoplasms, endoscopy, intestinal neoplasms

## Abstract

**Objectives:**

We previously reported a potential diagnostic algorithm for superficial non‐ampullary duodenal epithelial tumors (SNADETs) using white‐light magnifying endoscopy with crystal violet staining (ME‐CV). This study aimed to determine the diagnostic performance of the scheme and compare it with the conventional white‐light endoscopy (WLE) scoring system in a prospectively accumulated cohort.

**Methods:**

This was a single‐center prospective cohort study conducted over a 3‐year period. The primary endpoint was the diagnostic performance of ME‐CV in distinguishing Vienna classification (VCL) category 4/5 (C4/5) from category 3 (C3) SNADETs, including the positive predictive value (PPV) and negative predictive value (NPV), as evaluated by two expert endoscopists. We compared the diagnostic performance of the WLE and ME‐CV algorithms.

**Results:**

Fifty patients with SNADETs were enrolled. The inter‐observer agreement for the WLE scoring system and the ME‐CV algorithm was good (kappa 0.66 and 0.63). The PPV and NPV of the ME‐CV algorithm, when applied by expert endoscopists, were 25.6% and 90.2%, respectively. The sensitivity, specificity, PPV, NPV, and accuracy of the WLE scoring system were 43.8%, 73.8%, 24.1%, 87.3%, and 69.0%, respectively. The sensitivity, specificity, and accuracy of the ME‐CV algorithm were 62.5%, 65.5%, and 65.0%, respectively. Comparison of the diagnostic performance between the two systems demonstrated the significantly higher sensitivity of the ME‐CV algorithm (WLE, 43.8%; ME‐CV, 62.5%; *p* = 0.029).

**Conclusions:**

The ME‐CV algorithm had higher sensitivity than the WLE scoring system for distinguishing VCL C4/5 from VCL C3 in SNADETs, suggesting its potential to improve diagnosis and for selecting appropriate endoscopic resection.

## 1 Introduction

Superficial nonampullary duodenal tumors (SNADETs) are relatively rare [1]. However, it has recently been reported that the incidence of duodenal cancer in Japan is higher than that in Western countries [2]. More recently, the first Japanese guideline for the treatment of duodenal cancer has been published [3]. The guideline recommends endoscopic treatment for adenomas or duodenal cancers that are restricted to the mucosal layer. However, they do not mention the choice of the method of endoscopic resection (ER), presumably because of insufficient evidence in relation to SNADETs.

The efficacy and safety of novel ER methods for SNADETs, such as cold snare polypectomy (CSP) [4, 5] and underwater endoscopic mucosal resection (UEMR) [6, 7], have been reported. A recent randomized controlled trial demonstrated that UEMR was superior to CSP with respect to vertical resectability [8], suggesting that UEMR should be chosen for lesions with suspected submucosal involvement, whereas CSP is preferable for most small SNADETs. For large SNADETs, the treatment options include piecemeal EMR, endoscopic submucosal dissection (ESD), and surgical resection. Although ESD has high en bloc resection rates and favorable local resectability, it remains technically challenging [9, 10]. Accordingly, treatment strategies are currently determined at each institution based on factors such as lesion size and histological atypia.

Therefore, the preoperative endoscopic diagnosis of SNADETs, particularly the distinction of high‐grade adenoma/carcinoma (Vienna classification [VCL] category 4/5, [VCL C4/5]) from low‐grade adenoma (VCL category 3, [VCL C3]) [9, 11] is inevitable for the selection of an appropriate treatment method. However, a widely accepted algorithm for the endoscopic diagnosis of SNADETs has not yet been established [12].

We previously analyzed the findings obtained by magnifying endoscopy with crystal violet staining (ME‐CV) for SNADETs and proposed a diagnostic algorithm applicable to the distinction of VCL C4/5 from VCL C3 [13, 14, 15]. However, there seems to be a need for a prospective study to confirm the algorithm because our previous studies were retrospective. Furthermore, no prospective study has examined the accuracy of the endoscopic diagnosis of SNADETs. Therefore, we conducted a prospective cohort study to clarify the diagnostic performance of ME‐CV for SNADETs in comparison to white‐light endoscopy (WLE).

## 2 Methods

### 2.1 Study Design

This was a prospective cohort study conducted at Iwate Medical University from September 2021 to September 2024. The study protocol was approved by the Institutional Review Board of Iwate Medical University (No. MH2021‐077) and registered in the University Hospital Medical Network Clinical Trials Registry (UMIN000044679).

### 2.2 Patients

Consecutive patients referred to our hospital for the treatment of SNADETs or those diagnosed with SNADETs at our hospital during the study period were assessed for eligibility. The inclusion criteria were as follows: 1) patients suspected of having SNADETs by endoscopy, 2) ≥20 years of age at the time of enrollment, and 3) fully informed written consent for participation in the study (the patient or their surrogate). Bioptic confirmation of SNADETs was not an inclusion criterion. The exclusion criteria were 1) a diagnosis of familial adenomatous polyposis, 2) a remnant lesion after ER, and 3) patients deemed inappropriate for enrollment by the attending physician for other reasons. All patients provided their written informed consent to participate in the study.

### 2.3 Evaluation for Endoscopic Image of SNADETs

Each SNADET was observed under WLE conditions and subsequently evaluated using ME‐CV (GIF‐H290Z, Olympus, Tokyo, Japan). Tumor size was measured using an endoscopic measuring forceps (M2‐3U; Olympus, Tokyo, Japan). The ME‐CV findings were obtained in accordance with our previous reports. After spraying 0.05% crystal violet solution, endoscopic images were obtained at various magnifications with a maximum magnification of ×85. All endoscopic procedures were performed by an expert endoscopist (Yosuke Toya) and were recorded as videos for subsequent review. The endoscopic videos were reviewed in a blinded post hoc review by two experts (Shun Yamada and Masaki Endo) and two non‐experts (Tomo Kumei and Shun Yamada), with all prospectively collected cases, according to the study protocol.

**FIGURE 1:**
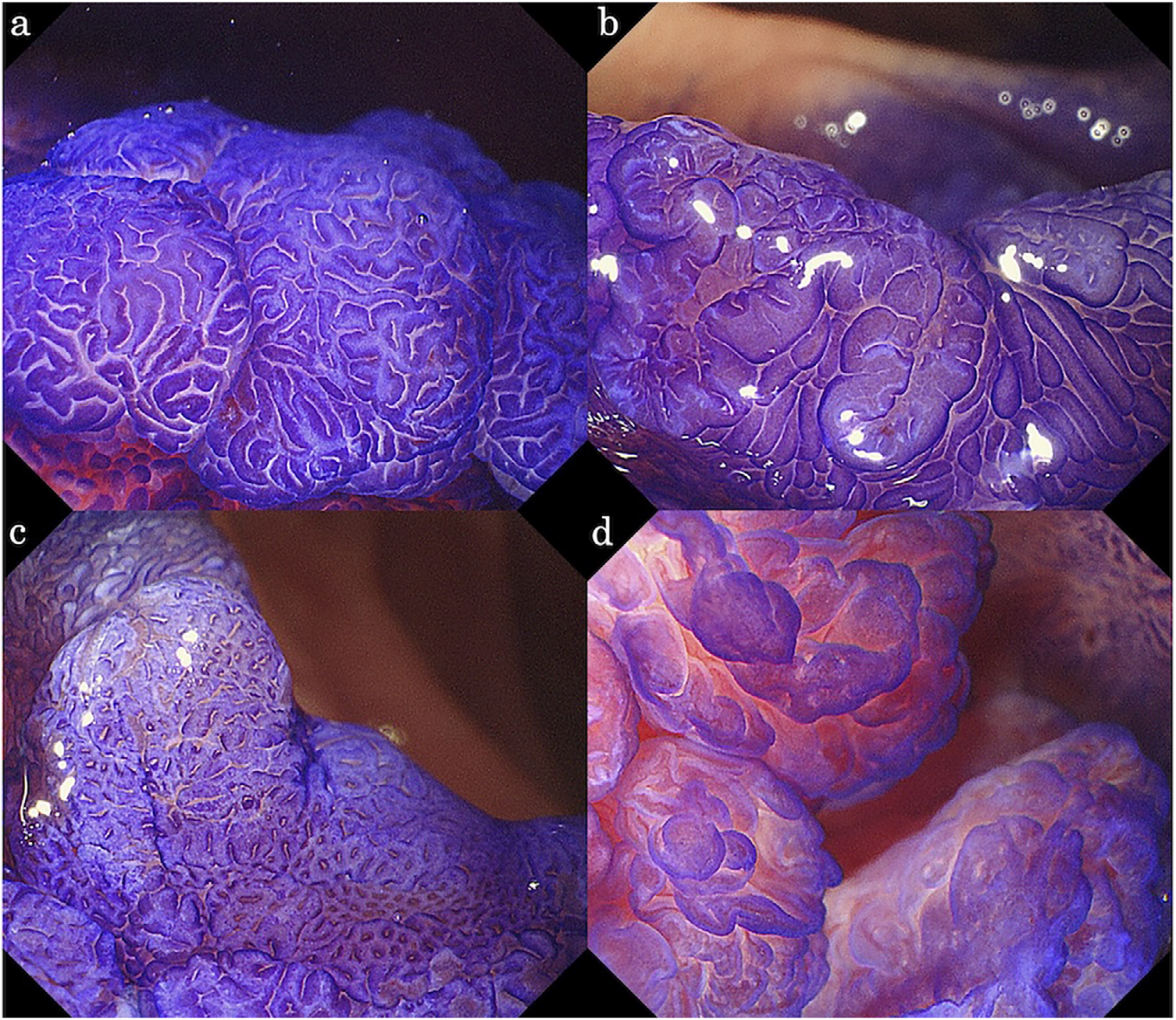
Endoscopic images of magnifying endoscopy with crystal violet staining (ME‐CV). (a) Convoluted pattern. (b) Leaf‐like pattern. (c) Reticular/sulciolar pattern. (d) Pinecone pattern.

**FIGURE 2:**
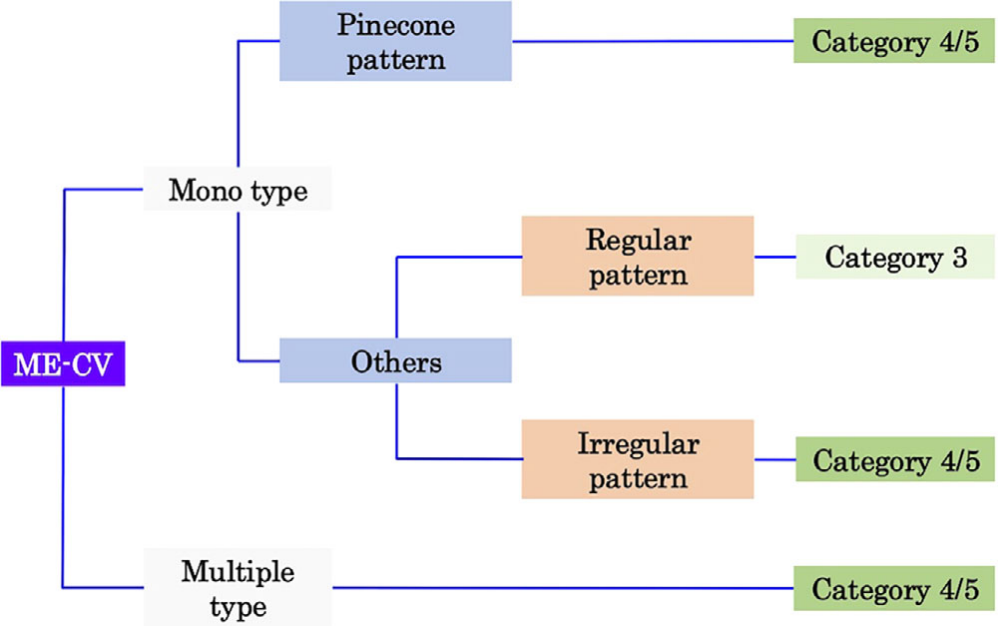
The magnifying endoscopy with crystal violet staining (ME‐CV) algorithm for superficial non‐ampullary duodenal epithelial tumors (SNADETs). Monotypes with a pinecone pattern according to the ME‐CV were classified as Vienna classification category 4/5. Except for the pinecone pattern, monotypes with a regular pattern were classified as category 3. Monotypes with irregular and multiple‐type surface patterns were classified as category 4/5.

The WLE images were assessed using the scoring system proposed by Kakushima et al. [16]. The system consists of endoscopic lesion size, color, macroscopic type, and nodularity (Table 1). A total score of ≥3 points was set as the cut‐off value for predicting VCL C4/5. The ME‐CV images were evaluated using a diagnostic algorithm for SNADETs. We have previously reported that the surface features of SNADETs under ME‐CV can be classified into convoluted, leaf‐like, reticular/sulciolar, and pinecone patterns (Figure 1) [13, 14]. Surface ME‐CV patterns were classified into pinecone, irregular, multiplicity, and monotonous patterns. The pinecone, irregular, and multiplicity patterns were regarded as suggestive of VCL C4/5, and the monotonous pattern as VCL C3 (Figure 2) [15].

**TABLE 1:** White light endoscopy scoring system.^[^ ^14^ ^]^

Endoscopic finding	Score		
	0	1	2
Lesion diameter	<10 mm	≥10 mm	
Color	White	Isochromatic	Red
Macroscopic type	Is, Ip, IIa without depression	Any type with depression or mixed type	
Nodularity	Uniform	Heterogeneous or none	

### 2.4 Pathological Examinations

All lesions were removed via ER or surgery and processed for pathological evaluations. Each lesion was graded histologically according to the VCL as C3 (low‐grade adenoma/dysplasia) or C4/5 (mucosal high‐grade neoplasia/submucosal carcinoma invasion) [9]. Immunohistochemical examinations were conducted using an autoimmunostaining system (Dako EnVision System, Denmark). Based on our previous report [14], the gastric type was defined as a phenotype positive for gastric markers only (MUC5AC and MUC6). Similarly, the intestinal type was defined as a phenotype that was positive for intestinal markers only (MUC2 and CD10). Tumors positive for both gastric and intestinal markers were regarded as a gastrointestinal type. All histological evaluations were performed by at least two of the four participating pathologists (Ryo Sugimoto, Noriyuki Uesugi, Tamotsu Sugai, and Naoki Yanagawa). Each pathologist independently assessed the specimens, and the final diagnoses were determined through discussion until a consensus was reached.

### 2.5 Outcomes

The primary endpoint was the diagnostic performance of the ME‐CV algorithm, including the positive predictive value (PPV) and negative predictive value (NPV), evaluated by two expert endoscopists. The expert endoscopists were board‐certified members of the Japanese Gastroenterological Endoscopy Society. The secondary endpoints included a comparison of the diagnostic performance, including sensitivity, specificity, PPV, NPV, and accuracy, between the WLE scoring system and the ME‐CV algorithm, a comparison of the diagnostic performance between the expert endoscopists and the non‐expert endoscopists, and the interobserver agreement for the WLE scoring system and the ME‐CV algorithm. In addition, we calculated the diagnostic performance of the combined use of WLE and ME‐CV, classifying lesions as VCL 4/5 when both methods indicated VCL 4/5.

### 2.6 Sample Size

Our previous study revealed that the diagnostic performances of the ME‐CV algorithm for distinguishing VCL C3 from C4/5 were as follows: sensitivity, 63.6%; specificity, 85.2%; PPV, 63.6%; NPV, 85.1% [14]. Assuming a PPV of 70% and a 30% prevalence of VCL C4/5, the required sample size was calculated using a normal approximation to ensure that the lower limit of the one‐sided 95% confidence interval (CI) for the PPV exceeded 50%. Consequently, the target sample size for this study was set at 50.

### 2.7 Statistical Analysis

Continuous variables were expressed as the median and interquartile range (IQR), and categorical variables were expressed as the number (%). The diagnostic performance of the WLE scoring system and ME‐CV algorithm, along with comparisons of the diagnostic performance among endoscopic methods and between experts and non‐experts, was analyzed using generalized estimating equations. In each analysis, *p*‐values of <0.05 were considered statistically significant. To evaluate the reproducibility of the endoscopic findings, the inter‐observer agreement for each lesion was calculated using a kappa analysis. All statistical data were analyzed using SAS (ver. 9.4; SAS Institute Inc., Cary, NC, USA). All statistical analyses were performed by an independent biostatistician (Fumiaki Takahashi).

## 3 Results

**FIGURE 3:**
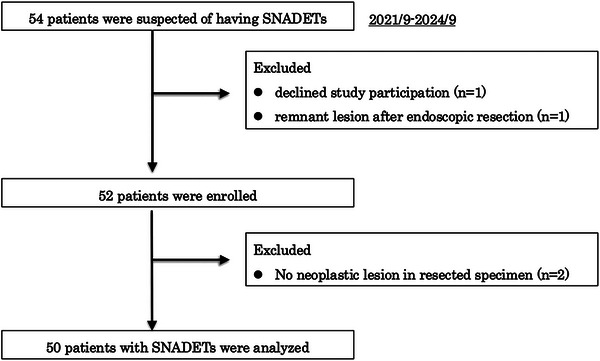
Patient flow in this study.

During the study period, 54 patients suspected of having SNADETs were recruited. After the exclusion of one patient who declined study participation and another with a remnant lesion after ER, 52 patients were enrolled. Two additional patients were excluded due to the absence of a neoplastic lesion in the resected specimen, leaving 50 patients for the final analyses (Figure 3).

The clinical characteristics of the patients are shown in Table 2. The median age was 70 years, with a male predominance (64.0%). The lesions were more frequently located on the oral side of the papilla of Vater (66.0%), with a median tumor size of 8.5 mm. A total of 41 lesions (82.0%) were of the elevated type, and most (96%) were treated with ER. Based on the VCL, 42 lesions (84%) were classified as VCL C3, and eight lesions (16.0%) were classified as VCL C4/5. Immunohistochemistry revealed 8 gastric type lesions (16.0%), 17 gastrointestinal type lesions (34.0%), and 25 intestinal type lesions (50%).

**TABLE 2:** Clinical characteristics of the study patients (*n* = 50).

Variable	
Age, years, median (IQR)	70 (60–75)
Sex, *n* (%)	
Male	32 (64.0)
Female	18 (36.0)
Tumor location, *n* (%)	
Oral side of the papilla of Vater	33 (66.0)
Anal side of the papilla of Vater	17 (34.0)
Tumor size, mm, median (IQR)	8.5 (5.8–14.0)
Macroscopic appearance, *n* (%)	
Elevated type	41 (82.0)
Depressed or Mixed type	9 (18.0)
Therapeutic method, *n* (%)	
Endoscopic resection (EMR or ESD)	48 (96.0)
Surgery	2 (4.0)
VCL, *n* (%)	
Category 3	42 (84.0)
Category 4/5	8 (16.0)
Mucin phenotype, *n* (%)	
Gastric type	8 (16.0)
Gastrointestinal type	17 (34.0)
Intestinal type	25 (50.0)

Abbreviations: EMR, endoscopic mucosal resection; ESD, endoscopic submucosal dissection; IQR, inter‐quartile range; VCL, Vienna Classification.

**FIGURE 4:**
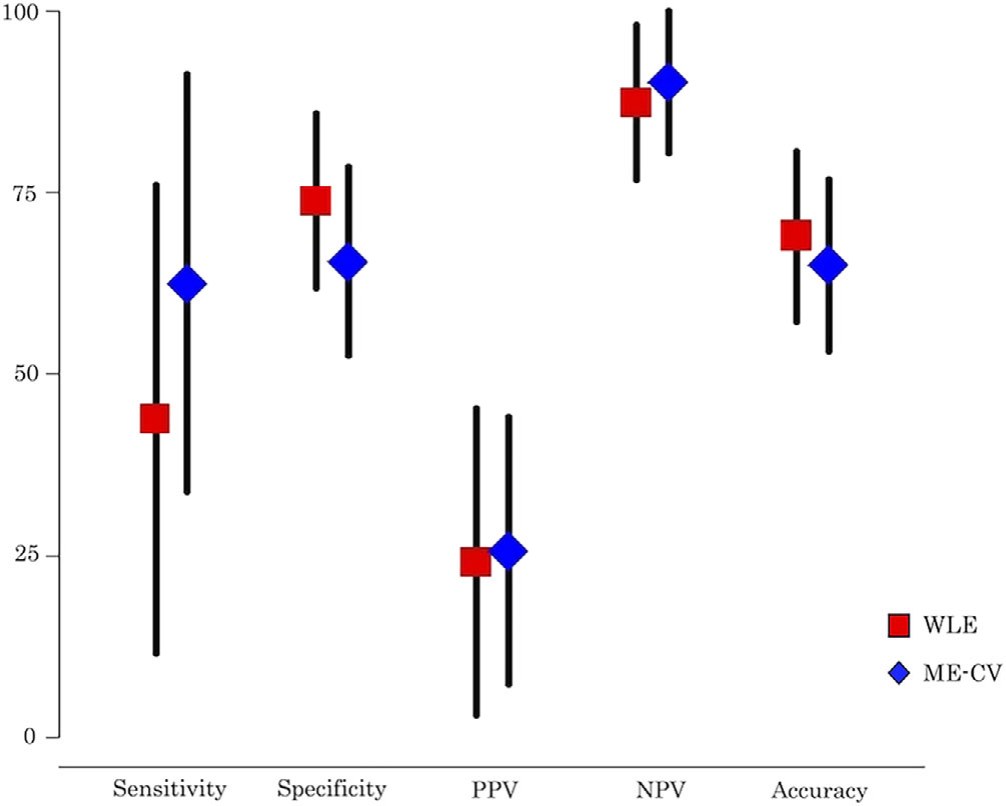
Diagnostic performance of the white light endoscopy (WLE) scoring system and the magnifying endoscopy with crystal violet staining (ME‐CV) algorithm by expert endoscopists.

Table 3 shows the diagnostic values of the WLE and ME‐CV algorithms for the expert and non‐expert endoscopists. The PPV and NPV of the ME‐CV algorithm by the expert endoscopists were 25.6% and 90.2%, respectively. The diagnostic performance of the WLE and the ME‐CV algorithms by the expert endoscopists is summarized in Figure 4 and Table 4. The sensitivity, specificity, PPV, NPV, and accuracy of the WLE scoring system were 43.8%, 73.8%, 24.1%, 87.3%, and 69.0%, respectively. The sensitivity, specificity, and accuracy of the ME‐CV algorithm were 62.5%, 65.5%, and 65.0%, respectively. When the diagnostic performance was compared between the WLE scoring system and the ME‐CV algorithm, the sensitivity was significantly higher in the latter than in the former (43.8% vs 62.5%; *p* = 0.029), whereas other diagnostic test results did not differ between the two procedures.

**TABLE 3:** Diagnostic value of the white light endoscopy (WLE) scoring system and the magnifying endoscopy with crystal violet staining (ME‐CV) algorithm for expert and non‐expert endoscopists.

	WLE scoring system			ME‐CV		
Expert 1	C3	C4/5	total	C3	C4/5	total
VCL						
C3	30	12	42	24	18	42
C4/5	4	4	8	3	5	8
total	34	16	50	27	23	50

Expert 2	C3	C4/5	total	C3	C4/5	total
VCL						
C3	32	10	42	31	11	42
C4/5	5	3	8	3	5	8
Total	37	13	50	34	16	50

Non‐expert 1	C3	C4/5	Total	C3	C4/5	Total
VCL						
C3	30	12	42	33	9	42
C4/5	5	3	8	2	6	8
Total	35	15	50	35	15	50

Non‐expert 2	C3	C4/5	Total	C3	C4/5	Total
VCL						
C3	28	14	42	17	25	42
C4/5	4	4	8	3	5	8
Total	32	18	50	20	30	50

Abbreviations: C3, category 3; C4/5, category 4/5; ME‐CV, magnifying endoscopy with crystal violet staining; VCL, Vienna Classification; WLE, white light endoscopy.

**TABLE 4:** Diagnostic performance of the white light endoscopy (WLE) scoring system and the magnifying endoscopy with crystal violet staining (ME‐CV) algorithm for expert endoscopists.

Modality	Sensitivity% (95% CI)	*p‐*Value	Specificity% (95% CI)	*p‐*Value	*p‐*Value	*p‐*Value	*p‐*Value	*p‐*Value	Accuracy% (95% CI)	*p‐*Value
WLE	43.8 (11.6–75.9)	0.029	73.8 (61.8–85.8)	0.18	24.1 (3.1–45.2)	0.78	87.3 (76.7–98.0)	0.20	69.0 (57.3–80.7)	0.48
ME‐CV	62.5 (33.8–91.2)		65.5 (52.5–78.5)		25.6 (7.3–44.0)		90.2 (80.5–99.9)		65.0 (53.2–76.8)	

Abbreviations: CI, confidence interval; ME‐CV, magnifying endoscopy with crystal violet staining; NPV, negative predictive value; PPV, positive predictive value; WLE, white light endoscopy.

Table 5 shows the diagnostic performance of the WLE and the ME‐CV algorithms for the expert and non‐expert endoscopists. The diagnostic test results for both procedures did not differ between expert and non‐expert endoscopists.

**TABLE 5:** Diagnostic performance of the WLE scoring system and the magnifying endoscopy with crystal violet staining (ME‐CV) algorithm or expert and non‐expert endoscopists.

Modality	Sensitivity% (95% CI)	*p‐*Value	*p‐*Value	*p‐*Value	*p‐*Value	*P*‐value	NPV % (95% CI)	*p‐*Value	Accuracy% (95%CI)	*p‐*Value
WLE (non‐expert)	43.8 (16.7–70.8)	1.0	69.1 (56.7–81.4)	0.31	21.2 (3.6–38.8)	0.63	86.6 (75.9–97.2)	0.77	65.0 (53.5–76.5)	0.37
WLE (expert)	43.8 (11.6–75.9)		73.8 (61.8–85.8)		24.1 (3.1–45.2)		87.3 (76.7–98.0)		69.0 (57.3–80.7)	
ME‐CV (non‐expert)	68.8 (39.1–98.4)	0.29	59.5 (49.0–70.1)	0.36	24.4 (7.7–41.2)	0.76	90.9 (80.7–101.1)	0.68	61.0 (50.9–71.1)	0.48
ME‐CV (expert)	62.5 (33.8–91.2)		65.5 (52.5–78.5)		25.6 (7.3–44.0)		90.2 (80.5–99.9)		65.0 (53.2–76.8)	

Abbreviations: CI, confidence interval; ME‐CV, magnifying endoscopy with crystal violet staining; NPV, negative predictive value; PPV, positive predictive value; WLE, white light endoscopy.

For experts, diagnostic accuracy differed between WLE and the combined use of WLE and ME‐CV. The diagnosis of VCL 4/5 by a combination of both WLE and ME‐CV showed higher specificity (80.9% vs 73.8%; *p* = 0.027), PPV (30.4% vs 24.1%; *p* = 0.03), and accuracy (75.0% vs 69%; *p* = 0.009) in comparison to WLE alone (Table S1).

The inter‐observer agreement between the two expert endoscopists was found to be good in the WLE scoring system (kappa value: 0.66) and in the ME‐CV algorithm (kappa value: 0.63). In contrast, the agreements between the non‐experts remained moderate (kappa value: 0.51 for the WLE scoring system, and 0.22 for the ME‐CV algorithm).

## 4 Discussion

A standard method for the endoscopic diagnosis of SNADETs remains to be fully established. Furthermore, most studies to date have reported on the value of endoscopy for the prediction of the histological grade of atypia in SNADETs and are retrospective in nature. To the best of our knowledge, this is the first prospective study to assess the diagnostic test value of ME‐CV in SNADETs. Our results demonstrated that the ME‐CV algorithm had significantly higher sensitivity than the WLE scoring system for distinguishing VCL C4/5 from VCL C3 SNADETs.

Several studies have reported on the use of magnifying image‐enhanced endoscopy (IEE) to diagnose the histological grade of SNADETs [12]. As an image‐enhancement tool, most reports have applied narrow‐band imaging (ME‐NBI) to target SNADETs, and the diagnostic test results ranged from 76% to 95.8% for sensitivity, from 53.4% to 97.4% for specificity, and from 65.1% to 96.8% for accuracy [17, 18, 19, 20]. In previous studies, an irregular micro surface pattern (MSP) or a combination of MSPs, rather than an irregular microvascular pattern (MVP), was preferentially used to indicate VCL C4/5. The lower diagnostic value of MVP has been explained by the obscured visualization of vasculature under NBI due to the prominent white opaque substance (WOS) in SNADETs^12^. Therefore, it seems reasonable to focus on the MSP for the diagnosis of SNADETs. Although less convenient than IEE, we believe that ME‐CV is another useful procedure to focus on the MSP of SNADETs. While previous studies have reported high overall diagnostic performance with ME‐NBI when compared to our ME‐CV results, such a difference may partly be a consequence of variations among study designs and the limited number of subjects in our study. A prospective head‐to‐head comparison between ME‐CV and ME‐NBI is warranted to clarify their relative diagnostic value.

We found that our ME‐CV algorithm was characterized by significantly higher sensitivity than the WLE scoring system for distinguishing VLC4/5 from VCL C3 in SNADETs. In contrast, there were no significant differences in the specificity, PPV, or NPV. This finding highlights a potential trade‐off in diagnostic yield. Although ME‐CV provides higher sensitivity than WLE and may help to reduce missed diagnoses of VLC4/5, its relatively lower specificity could increase the risk of false‐positive findings. Such a trend may result in unnecessary additional biopsies or ERs, raising the possibility of overtreatment. Therefore, the clinical application of ME‐CV should carefully balance these advantages and limitations.

A recent meta‐analysis reported that summary estimates of sensitivity, specificity, and area under the curve (AUC) for the endoscopic diagnosis of VCL4/5 were 80%, 80%, and 0.859, respectively, for WLE and 72%, 76%, and 0.811 for ME‐NBI [21]. These findings suggest that WLE may have comprehensive diagnostic potential for identifying severe atypia in SNADETs. In addition, a meta‐analysis showed that the combination of WLE and ME‐NBI yielded the highest sensitivity, specificity, and AUC values (88%, 87%, and 0.929, respectively) for the diagnosis of VCL4/5. Our results also showed a trend towards better diagnostic performance when the WLE and ME‐CV were combined, although this study was not designed to evaluate the diagnostic performance of the combination. Further investigation is warranted to establish and evaluate endoscopic diagnostic approaches that combine WLE and ME‐CV.

Our results did not show a significant difference in the diagnostic performance between the expert and the non‐expert groups for either WLE or ME‐CV. However, the kappa value for ME‐CV was extremely low in the non‐expert group (0.22). This observation suggests that the ME‐CV algorithm requires a certain level of proficiency and learning for the magnifying observation of gastric and colorectal lesions. Further studies are warranted to evaluate the impact of the learning curve on the accuracy of the ME‐CV algorithm in the diagnosis of SNADETs.

The present study had several limitations. First, it was a single‐center study. Since the attending endoscopists had experience in observing ME‐CV findings prior to the present study, they might have too much experience to generalize our findings worldwide. Second, the PPVs for both WLE and ME‐CV in this study were much lower than those reported in a previous retrospective study. This may be partly explained by the smaller number of VCL C4/5 SNADETs than initially anticipated. In addition, the small number of VCL 4/5 lesions may represent a potential limitation in terms of the statistical power. Thus, it is necessary to validate our findings in a larger‐scale, multicenter, prospective study. Third, the number of cases in each subgroup of the mucin phenotype was too small to conduct a reliable statistical analysis of the diagnostic performance. Finally, and most importantly, it should be noted that CV is recognized as a potent carcinogen in mammalian cells [22]. However, there is currently no evidence to suggest that exposure to extremely small topical amounts of CV increases the risk of malignant neoplasms.

In conclusion, the ME‐CV algorithm had significantly higher sensitivity than the WLE scoring system for distinguishing VCL4/5 from VCL C3 in SNADETs. Our ME‐CV algorithm may contribute to the diagnosis of VCL C4/5 lesions and the selection of an appropriate ER for SNADETs.

## Author Contributions

**Tomo Kumei**: conceptualization, data curation, investigation, and writing ‐ original draft preparation. **Yosuke Toya**: conceptualization, data curation, formal analysis, investigation, methodology, and writing ‐ original draft preparation. **Shun Yamada**: data curation and writing ‐ review & editing. **Makoto Eizuka**: data curation and writing ‐ review & editing. **Shunichi Yanai**: data curation and writing ‐ review & editing. **Masaki Endo**: conceptualization and writing ‐ review & editing. **Ryo Sugimoto**: investigation and writing ‐ review & editing. **Noriyuki Uesugi**: investigation and writing ‐ review & editing. **Tamotsu Sugai**: investigation and writing ‐ review & editing. **Naoki Yanagawa**: investigation and writing ‐ review & editing. **Fumiaki Takahashi**: formal analysis and writing ‐ review & editing. **Takayuki Matsumoto**: supervision and writing ‐ review & editing. All authors have read and approved the final version of the manuscript.

## Conflicts of Interest

Yosuke Toya is the associate editor of DEN Open. Takayuki Matsumoto is a faculty member at the Japanese Society of Gastrointestinal Endoscopy. Fumiaki Takahashi is the associate editor of Digestive Endoscopy. The other authors declare no conflicts of interest.

## Ethics Statement

**Approval of the research protocol by an institutional review board**: The study protocol was approved by the institutional review board of Iwate Medical University (No. MH2021‐077).

## Consent

All patients provided written informed consent for study participation.

## Clinical Trial Registration

This study was registered with the University Hospital Medical Network Clinical Trials Registry (UMIN000044679).

## Supporting information




**Table S1**: Diagnostic performance of the combination of the WLE scoring system and the ME‐CV algorithm by expert endoscopists.
