# Supplementary material for: Diagnostic Performance of Magnifying Endoscopy With Crystal Violet Staining for Superficial Non‐ampullary Duodenal Epithelial Tumors: A Single‐center Prospective Study
Source: DEN Open. 2025 Oct 12;6(1):e70223. doi: 10.1002/deo2.70223 (PMC12515707; doi:10.1002/deo2.70223)
Supplement: Supplementary file 1 — Table S1: Diagnostic performance of the combination of the WLE scoring system and the ME‐CV algorithm by expert endoscopists. [file DEO2-6-e70223-s001.docx]

| **Supplementary Table1.** Diagnostic performance of the combination of the WLE scoring system and the ME-CV algorithm by expert endoscopists | | | | | | | | | | |
| --- | --- | --- | --- | --- | --- | --- | --- | --- | --- | --- |
| Modality | Sensitivity  % (95% CI)) | *P* value | Specificity  % (95% CI)) | *P* value | PPV  % (95% CI)) | *P* value | NPV  % (95% CI)) | *P* value | Accuracy  % (95%CI) | *P* value |
| WLE  (expert) | 43.8  (11.6-75.9) | - | 73.8  (61.8-85.8) | 0.027 | 24.1  (3.1-45.2) | 0.03 | 87.3  (76.7-98.0) | 0.08 | 69.0  (57.3-80.7) | 0.009 |
| Combination  (expert) | 43.8  (11.6-75.9) |  | 80.9  (71.1-90.8) |  | 30.4  (5.6-55.3) |  | 88.3  (78.5-98.1) |  | 75.0  (64.5-85.5) |  |
| WLE, white light endoscopy; ME-CV, magnifying endoscopy with crystal violet staining; CI, confidence interval; PPV, positive predictive value; NPV, negative predictive value. | | | | | | | | | | |
